# *trans*-Dichloridobis(propane-1,3-diamine-κ^2^ *N*,*N*′)chromium(III) perchlorate

**DOI:** 10.1107/S1600536811006349

**Published:** 2011-02-26

**Authors:** Jong-Ha Choi, William Clegg

**Affiliations:** a Department of Chemistry, Andong National University, Andong 760-749, Republic of Korea; b School of Chemistry, Newcastle University, Newcastle upon Tyne NE1 7RU, England

## Abstract

In the title compound, [CrCl_2_(C_3_H_10_N_2_)_2_]ClO_4_, the Cr^III^ atom is coordinated equatorially by four N atoms of two propane-1,3-diamine (tn) ligands and axially by two mutually *trans* Cl atoms, thus displaying a slightly distorted octa­hedral geometry with no crystallographically imposed symmetry. The two six-membered chair chelate rings in the complex cation are in an *anti* conformation with respect to each other. The Cr—N bond lengths range from 2.0831 (18) to 2.0917 (19) Å, and the Cr—Cl bond lengths are 2.3148 (6) and 2.3135 (6) Å. The perchlorate anions have slightly distorted tetra­hedral geometries. Weak inter­molecular hydrogen bonds involving the tn ligand NH groups as donors, and chloride ligands and anion O atoms as acceptors are observed.

## Related literature

For the synthesis, see: Couldwell & House (1972 ▶); House (1970 ▶). For related structures, see: Choi *et al.* (2002 ▶, 2007 ▶, 2008 ▶, 2010 ▶); Vaughn & Rogers (1985 ▶); Kou *et al.* (2001 ▶). For tn ligand geometry, see: Vaughn (1981 ▶). For the standard Cambridge Structural Database description, see: Allen (2002 ▶).
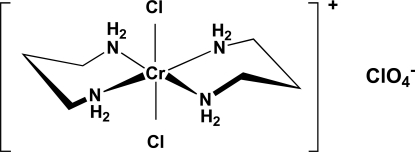


## Experimental

### 

#### Crystal data

[CrCl_2_(C_3_H_10_N_2_)_2_]ClO_4_
*M* *_r_* = 370.61
Monoclinic, 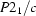

*a* = 6.4306 (5) Å
*b* = 17.2588 (15) Å
*c* = 13.0235 (11) Å
β = 92.840 (4)°
*V* = 1443.6 (2) Å^3^
*Z* = 4
Mo *K*α radiation
μ = 1.36 mm^−1^
*T* = 173 K
0.16 × 0.08 × 0.05 mm

#### Data collection

Bruker APEXII CCD diffractometer
Absorption correction: multi-scan (*TWINABS*; Sheldrick, 2008*a* ▶) *T*_min_ = 0.815, *T*_max_ = 0.930
28308 measured reflections
6269 independent reflections
5585 reflections with *I* > 2σ(*I*)
*R*_int_ = 0.039

#### Refinement

*R*[*F* ^2^ > 2σ(*F* ^2^)] = 0.028
*wR*(*F* ^2^) = 0.075
*S* = 1.07
6269 reflections
245 parameters
All H-atom parameters refined
Δρ_max_ = 0.36 e Å^−3^
Δρ_min_ = −0.32 e Å^−3^

### 

Data collection: *APEX2* (Bruker, 2010 ▶); cell refinement: *SAINT* (Bruker, 2010 ▶); data reduction: *SAINT*; program(s) used to solve structure: *SHELXTL* (Sheldrick, 2008*b* ▶); program(s) used to refine structure: *SHELXTL*; molecular graphics: *DIAMOND* (Brandenburg, 2010 ▶); software used to prepare material for publication: *SHELXTL* and local programs.

## Supplementary Material

Crystal structure: contains datablocks I, global. DOI: 10.1107/S1600536811006349/nk2086sup1.cif

Structure factors: contains datablocks I. DOI: 10.1107/S1600536811006349/nk2086Isup2.hkl

Additional supplementary materials:  crystallographic information; 3D view; checkCIF report

## Figures and Tables

**Table 1:** Hydrogen-bond geometry (Å, °)

*D*—H⋯*A*	*D*—H	H⋯*A*	*D*⋯*A*	*D*—H⋯*A*
N1—H1*A*⋯Cl1^i^	0.85 (3)	2.68 (3)	3.3684 (19)	139 (2)
N1—H1*B*⋯Cl1^ii^	0.89 (3)	2.77 (3)	3.5229 (19)	143 (2)
N2—H2*A*⋯O3^i^	0.81 (3)	2.45 (3)	3.182 (3)	151 (3)
N2—H2*A*⋯Cl2	0.81 (3)	2.67 (3)	3.072 (2)	112 (2)
N2—H2*B*⋯O4	0.86 (3)	2.35 (3)	3.134 (3)	151 (3)
N2—H2*B*⋯O2	0.86 (3)	2.56 (3)	3.326 (3)	149 (3)
N3—H3*A*⋯O2	0.87 (3)	2.15 (3)	3.000 (3)	166 (3)
N3—H3*B*⋯Cl2^iii^	0.89 (3)	2.58 (3)	3.3168 (19)	140 (2)
N4—H4*A*⋯O1^iv^	0.81 (3)	2.28 (3)	3.033 (3)	156 (3)

Symmetry codes: (i) 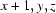; (ii) 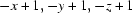; (iii) 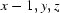; (iv) 
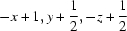
.

**Fig. 1.**
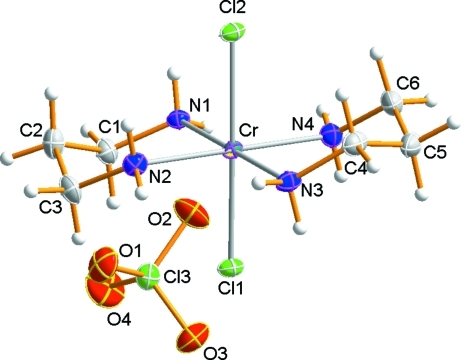
The structure of the complex cation and anion (displacement ellipsoids are drawn at the 50% probability level).

## supplementary crystallographic information

### Comment

The [Cr(tn)_2_*L*_2_]^+^ cation (tn = propane-1,3-diamine, *L* = monodentate ligand) can exist as *trans* and *cis* geometric isomers. There are also two possible conformations with respect to the six-membered chelate rings (present as chairs) in the *trans* geometric isomer: the carbon atoms of these rings in the two tn ligands can be located on the same side (*syn* conformer) or on opposite side (*anti* conformer) of the equatorial plane. In the crystal structures of *trans*-[Cr(Me_2_tn)_2_Cl_2_]Cl and *trans*-[Cr(Me_2_tn)_2_Br_2_]_2_Br_2_.HClO_4_.6H_2_O (Me_2_tn = 2,2-dimethylpropane-1,3-diamine), both *syn* and *anti* conformational isomers are found together (Choi *et al.*, 2002; Choi *et al.*, 2007), while *trans*-[Cr(Me_2_tn)_2_Cl_2_]ClO_4_ (Choi *et al.*, 2008) has only the *anti* conformer, as do *trans*-[Cr(tn)_2_F_2_]ClO_4_ (Vaughn & Rogers, 1985) and *trans*-[Cr(tn)_2_Cl_2_]_3_[Fe(CN)_6_.6H_2_O (Kou *et al.*, 2001). The preference for *syn* or *anti* conformation of chelate rings in *trans* complex cations with tn or Me_2_tn ligands is thus subtle and worthy of further study. Infrared and electronic absorption spectroscopic methods are not useful in distinguishing such *syn* and *anti* conformations in these metal complexes. Structural studies of bromido-containing chromium(III) complexes are relatively rare compared to those with chlorido ligands. Therefore we attempted to prepare *trans*-[Cr(tn)_2_Br_2_]ClO_4_ by a literature method (Couldwell & House, 1972); its UV-visible and IR spectra are nearly the same as those of *trans*-[Cr(tn)_2_Cl_2_]ClO_4_ (House, 1970), and it was only with a crystal structure analysis that we established that the product was actually the dichlorido rather than the dibromido complex. We report here the structure of *trans*-[Cr(tn)_2_Cl_2_]ClO_4_ (I) which provides further information on the conformation of the two six-membered chelate rings.

In the title complex (I), the chromium(III) ion is coplanar with the four coordinating N atoms and adopts an octahedral geometry, in which the four nitrogen atoms of two tn ligands occupy the equatorial sites and the two chlorine atoms coordinate axially in a *trans* configuration. The two six-membered rings have their usual stable chair conformations, and they are exclusively in the *anti* conformation with respect to each other in the unique cation of the asymmetric unit (Fig. 1).

The Cr—N distances (Table 1) are in the range 2.0831 (18)–2.0917 (19) Å, typical for Cr—N bonds involving primary amines (Choi *et al.*, 2002; Choi *et al.*, 2007). The Cr—Cl distances [2.3135 (6) and 2.3148 (6) Å] are very close to the values 2.3179 (9) and 2.3212 (4) Å found in *trans*-[Cr(Me_2_tn)_2_Cl_2_]ClO_4_ (Choi *et al.*, 2008), and typical generally of Cr—Cl bond lengths in the Cambridge Structural Database (Allen, 2002), but shorter than the 2.4743 (10) Å for Cr—Br bond lengths in *trans*-[Cr(en)_2_Br_2_]ClO_4_ (Choi *et al.*, 2010). The assignment of the axial ligands as Cl rather than the Br intended and expected from the synthesis is also clearly correct from the satisfactory refinement of anisotropic displacement parameters, demonstrating an appropriate electron density. The internal geometry of the tn ligands is typical for these in chair conformations (Vaughn, 1981). The uncoordinated ClO_4_^-^ anion shows an essentially tetrahedral arrangement with Cl—O distances in the range 1.4268 (19)–1.4380 (19) Å and the angles at Cl ranging from 108.32 (11) to 110.48 (13)°. There is an extensive weak hydrogen bonding network involving the oxygen atoms of the anions, chlorido ligands, and the N—H groups of the tn ligands (Table 2), which supports the main ionic interactions in this complex salt.

### Experimental

The ligand propane-1,3-diamine was obtained from Aldrich Chemical Co. and was used as supplied. All other chemicals were reagent grade materials and were used without further purification. We intended to prepare *trans*-[Cr(tn)_2_Br_2_]ClO_4_ as described in the literature (Couldwell & House, 1972) but obtained instead *trans*-[Cr(tn)_2_Cl_2_]ClO_4_, as demonstrated by this crystal structure analysis.

CrCl_3_.6H_2_O (5.4 g) was dissolved in DMSO (25 ml) and the solution was boiled for 10 min. A mixture of 1,3-propanediamine (3 ml) and DMSO (15 ml) was added and boiling was continued for 2 min. After cooling to 60°C, the solution was poured into well stirred acetone (300 ml). The precipitate was filtered off and washed with acetone, then dissolved in aqueous HBr (20 ml, 48%) and the solution was heated on a steam bath for 15 min. and filtered. The filtrate was heated on a steam bath for a further 15 min. Aqueous HClO_4_ (5 ml, 60%) was added to the solution. The resulting green crystals were collected and washed with ethanol. The infrared spectrum (nujol) was consistent with the crystallographically determined structure. The chloro ligands in the title compound are clearly retained from the chromium(III) chloride starting material, and were not substituted as intended by Br in the reaction with HBr.

### Refinement

The crystal was a non-merohedral twin with a 23.45 (6)% contribution of the minor component according to the refinement; because of the twinning, merging of symmetry-equivalent data could not be performed prior to refinement. The twin law is 1 0 0 / 0 - 1 0 / -0.2 0 - 1, corresponding to a 180° rotation about the *a* axis. Hydrogen atoms were located in a difference map and refined freely with individual isotropic displacement parameters.

### Crystal data

**Table:**

[CrCl_2_(C_3_H_10_N_2_)_2_]ClO_4_	*F*(000) = 764
*M**_r_* = 370.61	*D*_x_ = 1.705 Mg m^−^^3^
Monoclinic, *P*2_1_/*c*	Mo *K*α radiation, λ = 0.71073 Å
Hall symbol: -P 2ybc	Cell parameters from 6530 reflections
*a* = 6.4306 (5) Å	θ = 2.8–28.3°
*b* = 17.2588 (15) Å	µ = 1.36 mm^−^^1^
*c* = 13.0235 (11) Å	*T* = 173 K
β = 92.840 (4)°	Block, green
*V* = 1443.6 (2) Å^3^	0.16 × 0.08 × 0.05 mm
*Z* = 4	

### Data collection

**Table:**

Bruker APEXII CCD diffractometer	6269 independent reflections
Radiation source: sealed tube	5585 reflections with *I* > 2σ(*I*)
graphite	*R*_int_ = 0.039
Thin–slice ω scans	θ_max_ = 28.4°, θ_min_ = 2.0°
Absorption correction: multi-scan (TWINABS; Sheldrick, 2008*a*)	*h* = −8→8
*T*_min_ = 0.815, *T*_max_ = 0.930	*k* = 0→23
28308 measured reflections	*l* = 0→17

### Refinement

**Table:**

Refinement on *F*^2^	Secondary atom site location: difference Fourier map
Least-squares matrix: full	Hydrogen site location: difference Fourier map
*R*[*F*^2^ > 2σ(*F*^2^)] = 0.028	All H-atom parameters refined
*wR*(*F*^2^) = 0.075	*w* = 1/[σ^2^(*F*_o_^2^) + (0.0391*P*)^2^ + 1.9009*P*] where *P* = (*F*_o_^2^ + 2*F*_c_^2^)/3
*S* = 1.07	(Δ/σ)_max_ = 0.001
6269 reflections	Δρ_max_ = 0.36 e Å^−^^3^
245 parameters	Δρ_min_ = −0.32 e Å^−^^3^
0 restraints	Extinction correction: *SHELXTL* (Sheldrick, 2008a), Fc^*^=kFc[1+0.001xFc^2^λ^3^/sin(2θ)]^-1/4^
Primary atom site location: structure-invariant direct methods	Extinction coefficient: 0.0011 (6)

### Special details

**Table:**

Geometry. All e.s.d.'s (except the e.s.d. in the dihedral angle between two l.s. planes) are estimated using the full covariance matrix. The cell e.s.d.'s are taken into account individually in the estimation of e.s.d.'s in distances, angles and torsion angles; correlations between e.s.d.'s in cell parameters are only used when they are defined by crystal symmetry. An approximate (isotropic) treatment of cell e.s.d.'s is used for estimating e.s.d.'s involving l.s. planes.
Refinement. Refinement of *F*^2^ against ALL reflections. The weighted *R*-factor *wR* and goodness of fit *S* are based on *F*^2^, conventional *R*-factors *R* are based on *F*, with *F* set to zero for negative *F*^2^. The threshold expression of *F*^2^ > σ(*F*^2^) is used only for calculating *R*-factors(gt) *etc*. and is not relevant to the choice of reflections for refinement. *R*-factors based on *F*^2^ are statistically about twice as large as those based on *F*, and *R*- factors based on ALL data will be even larger.

### Fractional atomic coordinates and isotropic or equivalent isotropic displacement parameters (Å^2^)

**Table:**

	*x*	*y*	*z*	*U*_iso_*/*U*_eq_	
Cr	0.49360 (5)	0.396455 (18)	0.29650 (2)	0.01236 (9)	
Cl1	0.24916 (7)	0.41493 (3)	0.41882 (4)	0.01906 (12)	
Cl2	0.74177 (7)	0.37755 (3)	0.17627 (4)	0.01921 (12)	
N1	0.7262 (3)	0.42985 (10)	0.40534 (14)	0.0149 (3)	
H1A	0.840 (4)	0.4296 (16)	0.375 (2)	0.026 (7)*	
H1B	0.707 (4)	0.4791 (15)	0.4214 (19)	0.015 (6)*	
N2	0.5301 (3)	0.28012 (11)	0.33866 (15)	0.0177 (4)	
H2A	0.619 (5)	0.2656 (17)	0.301 (2)	0.028 (8)*	
H2B	0.417 (5)	0.2552 (18)	0.321 (2)	0.039 (8)*	
N3	0.2572 (3)	0.36594 (12)	0.18892 (14)	0.0182 (4)	
H3A	0.258 (4)	0.3159 (17)	0.182 (2)	0.027 (7)*	
H3B	0.138 (5)	0.3757 (16)	0.219 (2)	0.030 (8)*	
N4	0.4523 (3)	0.51159 (11)	0.25055 (15)	0.0194 (4)	
H4A	0.556 (4)	0.5348 (16)	0.271 (2)	0.024 (7)*	
H4B	0.356 (5)	0.5272 (16)	0.284 (2)	0.029 (8)*	
C1	0.7561 (3)	0.38579 (13)	0.50303 (17)	0.0190 (4)	
H1C	0.872 (4)	0.4054 (15)	0.543 (2)	0.022 (7)*	
H1D	0.634 (4)	0.3944 (13)	0.5441 (19)	0.011 (6)*	
C2	0.7850 (4)	0.29977 (13)	0.48554 (19)	0.0236 (5)	
H2C	0.894 (4)	0.2925 (15)	0.439 (2)	0.022 (7)*	
H2D	0.821 (4)	0.2755 (17)	0.550 (2)	0.034 (8)*	
C3	0.5888 (4)	0.25891 (13)	0.44656 (18)	0.0222 (5)	
H3C	0.605 (4)	0.2040 (16)	0.450 (2)	0.024 (7)*	
H3D	0.476 (4)	0.2728 (15)	0.490 (2)	0.023 (7)*	
C4	0.2446 (4)	0.40246 (14)	0.08533 (18)	0.0232 (5)	
H4C	0.366 (4)	0.3889 (15)	0.051 (2)	0.022 (7)*	
H4D	0.121 (4)	0.3793 (15)	0.045 (2)	0.021 (6)*	
C5	0.2255 (4)	0.48944 (15)	0.09247 (19)	0.0244 (5)	
H5C	0.203 (4)	0.5077 (16)	0.021 (2)	0.032 (8)*	
H5D	0.104 (4)	0.5017 (16)	0.129 (2)	0.029 (7)*	
C6	0.4160 (4)	0.52997 (14)	0.13932 (19)	0.0242 (5)	
H6C	0.542 (4)	0.5136 (15)	0.106 (2)	0.021 (6)*	
H6D	0.403 (4)	0.5848 (17)	0.135 (2)	0.029 (7)*	
Cl3	0.07592 (8)	0.14746 (3)	0.25004 (4)	0.01984 (12)	
O1	0.1011 (3)	0.06757 (10)	0.22292 (16)	0.0363 (4)	
O2	0.1833 (3)	0.19442 (11)	0.17838 (16)	0.0384 (5)	
O3	−0.1425 (3)	0.16517 (11)	0.24445 (15)	0.0358 (4)	
O4	0.1632 (3)	0.16154 (14)	0.35117 (15)	0.0475 (5)	

### Atomic displacement parameters (Å^2^)

**Table:**

	*U*^11^	*U*^22^	*U*^33^	*U*^12^	*U*^13^	*U*^23^
Cr	0.00998 (15)	0.01357 (16)	0.01352 (17)	−0.00015 (11)	0.00047 (11)	−0.00070 (12)
Cl1	0.0142 (2)	0.0238 (3)	0.0196 (3)	0.00007 (18)	0.00468 (18)	−0.00218 (19)
Cl2	0.0131 (2)	0.0257 (3)	0.0190 (3)	0.00135 (18)	0.00347 (18)	−0.00246 (19)
N1	0.0135 (8)	0.0154 (9)	0.0160 (9)	−0.0014 (6)	0.0014 (6)	−0.0011 (7)
N2	0.0171 (8)	0.0166 (9)	0.0192 (9)	−0.0015 (7)	−0.0009 (7)	−0.0017 (7)
N3	0.0133 (8)	0.0238 (10)	0.0175 (9)	−0.0010 (7)	−0.0003 (7)	−0.0028 (7)
N4	0.0197 (9)	0.0179 (9)	0.0206 (10)	0.0006 (7)	0.0006 (8)	0.0014 (7)
C1	0.0222 (10)	0.0194 (10)	0.0150 (10)	−0.0019 (8)	−0.0024 (8)	−0.0007 (8)
C2	0.0281 (12)	0.0194 (11)	0.0226 (12)	0.0020 (9)	−0.0065 (10)	0.0011 (9)
C3	0.0310 (12)	0.0155 (10)	0.0201 (11)	−0.0035 (9)	−0.0004 (9)	0.0030 (8)
C4	0.0191 (10)	0.0338 (13)	0.0164 (11)	0.0023 (9)	−0.0017 (8)	−0.0017 (9)
C5	0.0210 (11)	0.0329 (13)	0.0193 (11)	0.0081 (9)	0.0005 (9)	0.0048 (9)
C6	0.0269 (11)	0.0243 (12)	0.0216 (11)	0.0040 (9)	0.0031 (9)	0.0080 (9)
Cl3	0.0206 (2)	0.0175 (2)	0.0210 (3)	−0.00116 (19)	−0.00290 (19)	−0.00153 (19)
O1	0.0380 (10)	0.0179 (8)	0.0530 (13)	0.0042 (7)	0.0012 (9)	−0.0030 (8)
O2	0.0452 (11)	0.0343 (10)	0.0364 (11)	−0.0138 (9)	0.0092 (9)	0.0050 (8)
O3	0.0246 (9)	0.0394 (10)	0.0434 (12)	0.0087 (8)	0.0019 (8)	−0.0052 (9)
O4	0.0523 (13)	0.0653 (15)	0.0235 (10)	−0.0131 (11)	−0.0121 (9)	−0.0053 (10)

### Geometric parameters (Å, °)

**Table:**

Cr—Cl1	2.3148 (6)	C1—H1D	0.98 (2)
Cr—Cl2	2.3135 (6)	C1—C2	1.515 (3)
Cr—N1	2.0903 (18)	C2—H2C	0.95 (3)
Cr—N2	2.0917 (19)	C2—H2D	0.96 (3)
Cr—N3	2.0831 (18)	C2—C3	1.511 (3)
Cr—N4	2.0884 (19)	C3—H3C	0.95 (3)
N1—H1A	0.85 (3)	C3—H3D	0.97 (3)
N1—H1B	0.89 (3)	C4—H4C	0.95 (3)
N1—C1	1.486 (3)	C4—H4D	1.02 (3)
N2—H2A	0.81 (3)	C4—C5	1.510 (3)
N2—H2B	0.86 (3)	C5—H5C	0.99 (3)
N2—C3	1.483 (3)	C5—H5D	0.95 (3)
N3—H3A	0.87 (3)	C5—C6	1.513 (3)
N3—H3B	0.89 (3)	C6—H6C	0.98 (3)
N3—C4	1.488 (3)	C6—H6D	0.95 (3)
N4—H4A	0.81 (3)	Cl3—O1	1.4346 (18)
N4—H4B	0.82 (3)	Cl3—O2	1.4380 (19)
N4—C6	1.490 (3)	Cl3—O3	1.4360 (18)
C1—H1C	0.95 (3)	Cl3—O4	1.4268 (19)
			
Cl1—Cr—Cl2	179.11 (2)	N1—C1—C2	112.60 (19)
Cl1—Cr—N1	89.02 (5)	H1C—C1—H1D	106 (2)
Cl1—Cr—N2	91.31 (6)	H1C—C1—C2	109.5 (15)
Cl1—Cr—N3	90.00 (6)	H1D—C1—C2	109.7 (13)
Cl1—Cr—N4	89.14 (6)	C1—C2—H2C	108.8 (16)
Cl2—Cr—N1	90.19 (5)	C1—C2—H2D	108.8 (18)
Cl2—Cr—N2	88.28 (6)	C1—C2—C3	113.6 (2)
Cl2—Cr—N3	90.80 (6)	H2C—C2—H2D	110 (2)
Cl2—Cr—N4	91.29 (6)	H2C—C2—C3	110.8 (16)
N1—Cr—N2	91.11 (7)	H2D—C2—C3	104.7 (17)
N1—Cr—N3	178.42 (8)	N2—C3—C2	111.85 (19)
N1—Cr—N4	90.49 (7)	N2—C3—H3C	108.4 (16)
N2—Cr—N3	90.15 (8)	N2—C3—H3D	108.9 (16)
N2—Cr—N4	178.34 (8)	C2—C3—H3C	111.0 (16)
N3—Cr—N4	88.26 (8)	C2—C3—H3D	109.1 (16)
Cr—N1—H1A	106.5 (19)	H3C—C3—H3D	107 (2)
Cr—N1—H1B	108.7 (16)	N3—C4—H4C	108.4 (16)
Cr—N1—C1	119.81 (13)	N3—C4—H4D	108.1 (15)
H1A—N1—H1B	104 (2)	N3—C4—C5	111.49 (19)
H1A—N1—C1	108.7 (19)	H4C—C4—H4D	107 (2)
H1B—N1—C1	107.6 (16)	H4C—C4—C5	110.2 (16)
Cr—N2—H2A	102 (2)	H4D—C4—C5	111.1 (14)
Cr—N2—H2B	109 (2)	C4—C5—H5C	105.6 (16)
Cr—N2—C3	120.38 (14)	C4—C5—H5D	108.8 (17)
H2A—N2—H2B	107 (3)	C4—C5—C6	114.71 (19)
H2A—N2—C3	110 (2)	H5C—C5—H5D	108 (2)
H2B—N2—C3	108 (2)	H5C—C5—C6	108.0 (16)
Cr—N3—H3A	108.3 (19)	H5D—C5—C6	111.2 (17)
Cr—N3—H3B	105.8 (19)	N4—C6—C5	112.24 (19)
Cr—N3—C4	120.53 (14)	N4—C6—H6C	106.3 (15)
H3A—N3—H3B	104 (3)	N4—C6—H6D	106.0 (17)
H3A—N3—C4	109.2 (19)	C5—C6—H6C	110.9 (15)
H3B—N3—C4	107.9 (19)	C5—C6—H6D	111.8 (17)
Cr—N4—H4A	107 (2)	H6C—C6—H6D	109 (2)
Cr—N4—H4B	104 (2)	O1—Cl3—O2	108.55 (12)
Cr—N4—C6	119.53 (15)	O1—Cl3—O3	108.32 (11)
H4A—N4—H4B	107 (3)	O1—Cl3—O4	110.29 (13)
H4A—N4—C6	108 (2)	O2—Cl3—O3	110.30 (12)
H4B—N4—C6	111 (2)	O2—Cl3—O4	108.88 (13)
N1—C1—H1C	110.3 (16)	O3—Cl3—O4	110.48 (13)
N1—C1—H1D	108.4 (14)		
			
Cl1—Cr—N1—C1	59.00 (15)	Cl1—Cr—N4—C6	−130.76 (17)
Cl2—Cr—N1—C1	−120.57 (15)	Cl2—Cr—N4—C6	50.02 (17)
N2—Cr—N1—C1	−32.28 (16)	N1—Cr—N4—C6	140.22 (17)
N4—Cr—N1—C1	148.14 (16)	N3—Cr—N4—C6	−40.74 (17)
Cl1—Cr—N2—C3	−56.16 (16)	Cr—N1—C1—C2	53.7 (2)
Cl2—Cr—N2—C3	123.04 (16)	N1—C1—C2—C3	−71.2 (3)
N1—Cr—N2—C3	32.88 (17)	Cr—N2—C3—C2	−54.3 (2)
N3—Cr—N2—C3	−146.17 (17)	C1—C2—C3—N2	71.1 (3)
Cl1—Cr—N3—C4	130.33 (16)	Cr—N3—C4—C5	−58.4 (2)
Cl2—Cr—N3—C4	−50.08 (16)	N3—C4—C5—C6	67.0 (3)
N2—Cr—N3—C4	−138.36 (17)	Cr—N4—C6—C5	58.4 (2)
N4—Cr—N3—C4	41.19 (17)	C4—C5—C6—N4	−67.7 (3)

### Hydrogen-bond geometry (Å, °)

**Table:**

*D*—H···*A*	*D*—H	H···*A*	*D*···*A*	*D*—H···*A*
N1—H1A···Cl1^i^	0.85 (3)	2.68 (3)	3.3684 (19)	139 (2)
N1—H1B···Cl1^ii^	0.89 (3)	2.77 (3)	3.5229 (19)	143 (2)
N2—H2A···O3^i^	0.81 (3)	2.45 (3)	3.182 (3)	151 (3)
N2—H2A···Cl2	0.81 (3)	2.67 (3)	3.072 (2)	112 (2)
N2—H2B···O4	0.86 (3)	2.35 (3)	3.134 (3)	151 (3)
N2—H2B···O2	0.86 (3)	2.56 (3)	3.326 (3)	149 (3)
N3—H3A···O2	0.87 (3)	2.15 (3)	3.000 (3)	166 (3)
N3—H3B···Cl2^iii^	0.89 (3)	2.58 (3)	3.3168 (19)	140 (2)
N4—H4A···O1^iv^	0.81 (3)	2.28 (3)	3.033 (3)	156 (3)

Symmetry codes: (i) *x*+1, *y*, *z*; (ii) −*x*+1, −*y*+1, −*z*+1; (iii) *x*−1, *y*, *z*; (iv) −*x*+1, *y*+1/2, −*z*+1/2.
